# China's top 10 science and technology stories in 2021

**DOI:** 10.1093/nsr/nwac070

**Published:** 2022-04-05

**Authors:** Ling Xin

A list of the top 10 domestic science and technology stories was jointly released by the Chinese Academy of Sciences and the Chinese Academy of Engineering in Beijing on 18 January 2022. The stories ranged from space exploration to life sciences research to quantum technologies, and were selected by academicians from the two academies. It was the 28th time the list had been released.

## 1 SUCCESSFUL COMPLETION OF THE MARS MISSION

On 11 June 2021, the China National Space Administration revealed the first batch of images taken by the country's Mars rover Zhurong, including a selfie of the rover with the lander and a 360-degree panoramic view of the landing site, which marked the successful completion of China's first-ever mission to the red planet. The Tianwen-1 mission lifted off from Wenchang space launch center in July 2020 and arrived in Mars orbit the following February. On 14 May, Zhurong and the lander separated from the Tianwen-1 orbiter, and touched down successfully on the planned landing area of Utopia Planitia.

**Figure 1:**
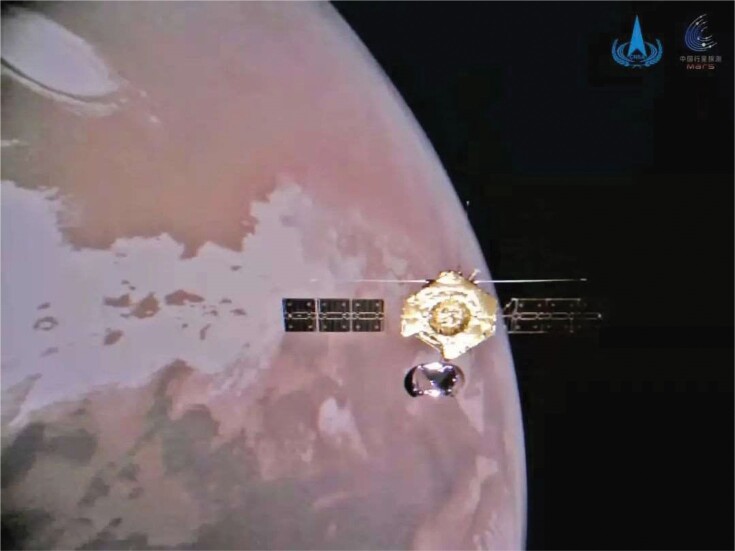
Tianwen-1, China's first mission to Mars, was decalred a success. (Courtesy of China National Space Administration)

## 2 CONTINUOUS HUMAN PRESENCE IN EARTH ORBIT

As a key step towards building its own space station, China launched two crewed spacecraft, Shenzhou 12 and Shenzhou 13, in June and October, respectively, sending a total of six astronauts to the space station's Tianhe core module in low Earth orbit. The first three-person shift stayed in orbit for three months and carried out spacewalks, robotic arm operations, scientific experiments and technological tests for long-term residence in space. The second shift, which included a female astronaut, were tasked with more activities, equipment tests and installations, and live science lectures. Their stay in orbit will be six months, until April 2022.

**Figure 2:**
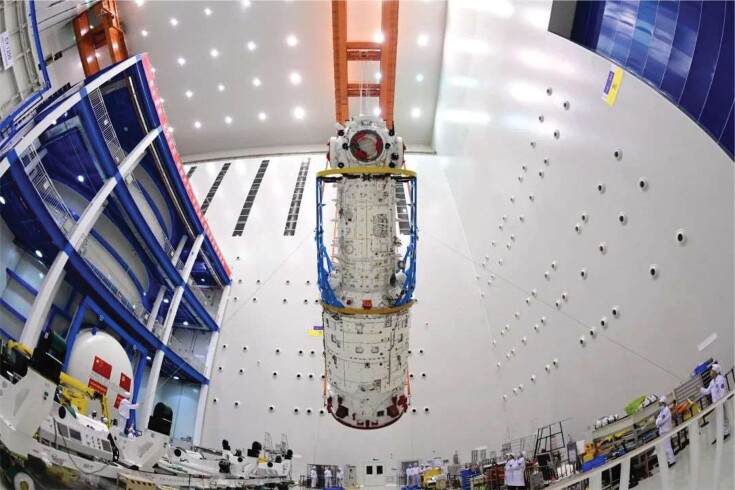
Tianhe, the core module of the China Space Station. (*Courtesy of the China Academy of Space Technology*)

## 3 SYNTHESIS OF STARCH FROM CO_2_

For the first time, researchers from the Tianjin Institute of Industrial Biotechnology, Chinese Academy of Sciences, used carbon dioxide to successfully synthesize starch in the laboratory. In a paper published in *Science* magazine, they adopted a building-block strategy based on chemical and biological catalytic modules to develop an 11-step approach to producing starch, which is much simpler and more efficient than the natural synthesis of starch in green plants. Given the essential role starch plays in our daily life and in industrial production, the research may have major implications for future biomanufacturing and food security.

**Figure 3:**
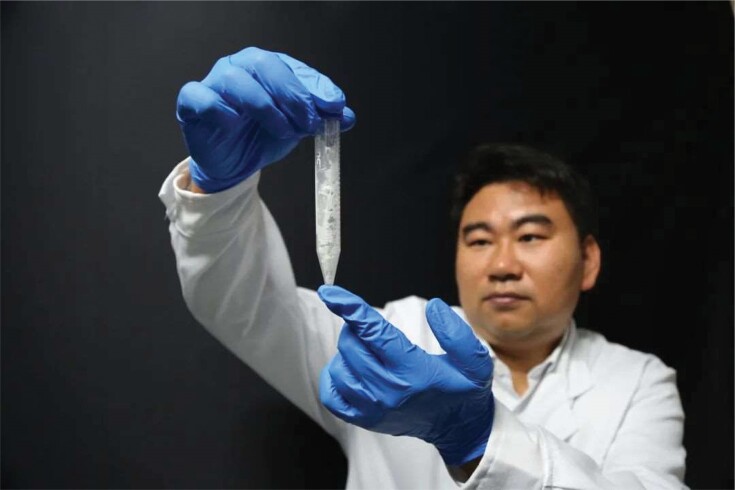
Starch synthesized from CO_2_. (*Courtesy of the Tianjin Institute of Industrial Biotechnology, Chinese Academy of Sciences*)

## 4 SUPERCOMPUTER VS. QUANTUM SUPREMACY

A team of 14 Chinese researchers took home the 2021 Association for Computing Machinery's Gordon Bell Prize for using a new exaflop-scale Sunway supercomputer to ‘achieve real-time simulation of a random quantum circuit and close the “quantum supremacy” gap.’ By introducing a systematic design process that covers the algorithm, parallelization and architecture required for the simulation, the team was able to simulate a 10 × 10 × (1 + 40 + 1) circuit in 304 seconds. It seriously challenged Google's claim that some complex problems would take a classical computer 10 000 years to solve, while the company's quantum processor Sycamore only needed 200 seconds. This prize-winning work may bring algorithmic and architectural innovation to the supercomputing community and speed up the development of quantum devices in the future.

**Figure 4:**
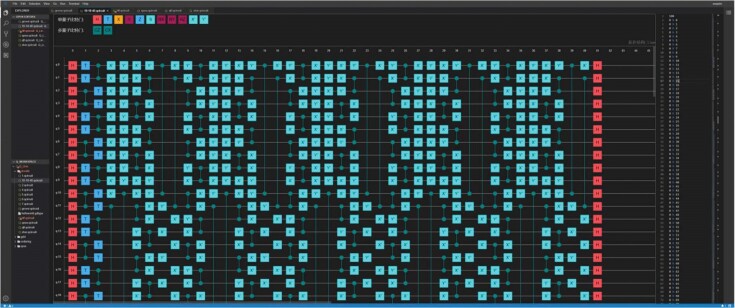
The user interface of the quantum cloud platform. (*Courtesy of the Zhejiang Lab and National Supercomputing Center Wuxi*)

## 5 HIGHEST ENERGY LIGHT FROM SPACE

An experiment located 4410 m above sea level on the Tibetan Plateau spotted the most energetic light particles ever observed by humans. According to a paper published in *Nature*, the Large High-Altitude Air Shower Observatory (LHAASO) used four different types of detectors to catch gamma ray photons up to 1.4 petaelectronvolts, which is a hundred times more energetic than what can be created in human-made colliders on Earth. Researchers traced the photons back to a dozen likely origins in the Milky Way Galaxy, which will help us better understand where exactly these rays come from and how they are produced.

**Figure 5:**
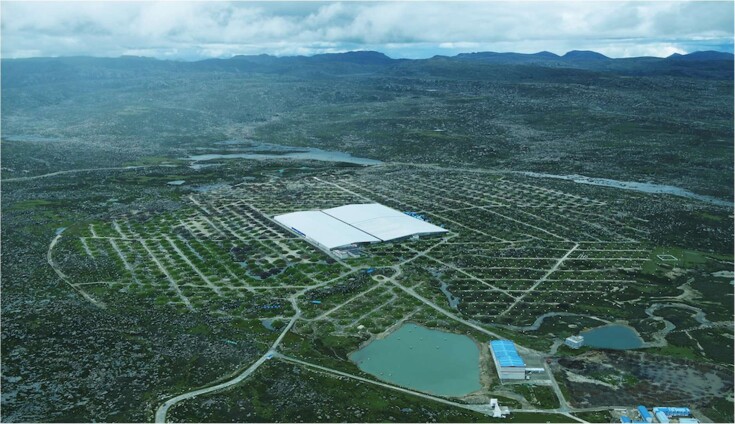
The construction of LHAASO was completed in July 2021. (*Courtesy of the Institute of High Energy Physics, Chinese Academy of Sciences*)

## 6 MOON ROCKS RESEARCH BONANZA

Using rock samples returned by China's Chang’e-5 lunar mission, researchers published a series of findings regarding the geological age and chemical composition of the sampling site. They measured the radioactive decay of isotopes in 47 hardened lava samples and dated them to 2.03 billion years old, which is ∼1 billion years younger than the rocks returned by US Apollo missions. Researchers also found that contrary to previous beliefs, the mantle source of the samples is depleted of heat-generating elements such as potassium, uranium and thorium, adding to the mystery of why the area was still active long after volcanic eruptions were thought to have stopped on the Moon.

**Figure 6:**
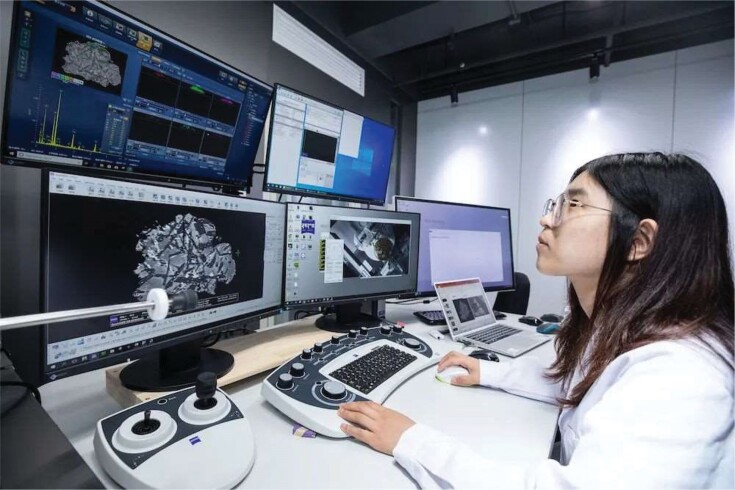
The researcher is analyzing the Chang’e-5 Moon sample with a scanning electron microscope. (*Courtesy of the Institute of Geology and Geophysics, Chinese Academy of Sciences*)

## 7 A NEW WAY TO BREED RICE

A group from the Institute of Genetics and Developmental Biology, Chinese Academy of Sciences, proposed a novel way to breed high-yielding rice through re-domestication. While all cultivated rice is diploid with two sets of chromosomes in each cell, scientists have long desired to grow polyploid rice, which comes with high nutrients, strong vitality and adaptability, among other advantages. The group first identified a wild allotetraploid rice variety, and then established a workable four-step route to domesticating it. Their study, published in *Cell*, showed a promising way to develop domesticated polyploid rice into a new staple cereal and help address the global food security challenge. 

**Figure 7:**
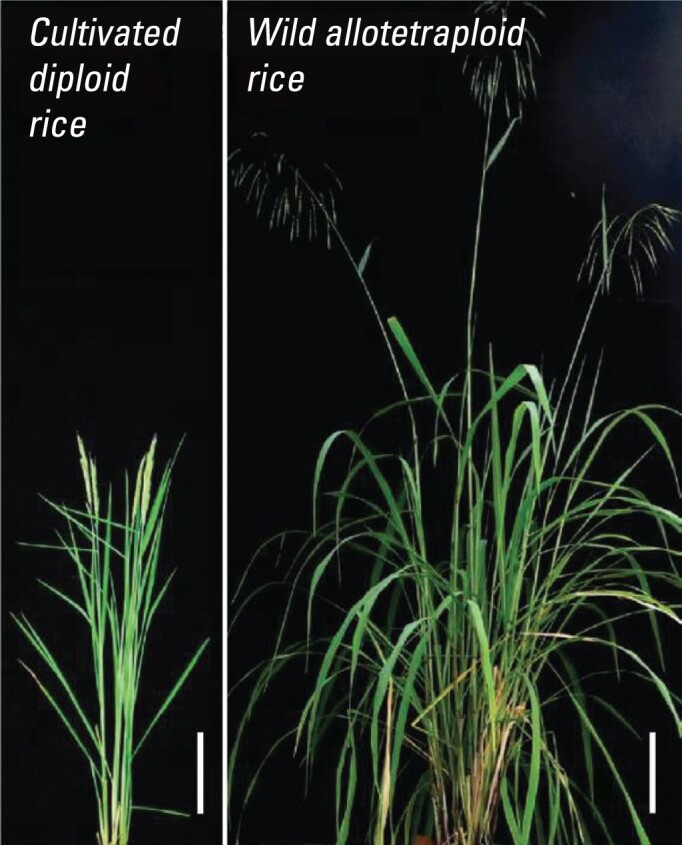
The whole plants of the cultivated diploid rice and the wild allotetraploid rice. Scale bars: 20 cm. *(Courtesy of the Institute of Genetics and Developmental Biology, Innovation Academy for Seed Design, Chinese Academy of Sciences)*

## 8 LARGE-SCALE HELIUM REFRIGERATION

In April, a major infrastructure project on ‘the development of a large-scale cryogenic refrigeration system in the liquid helium to superfluid helium temperature range’ passed national acceptance review. Led by researchers from the Technical Institute of Physics and Chemistry, Chinese Academy of Sciences, the project has enabled China to build kilowatt-level refrigeration equipment at -269°C and hundred-watt-level refrigeration equipment at -271°C, respectively, to answer the country's urgent needs in aerospace, basic research, hydrogen storage and transportation, as well as in a number of strategically important technologies.

**Figure 8:**
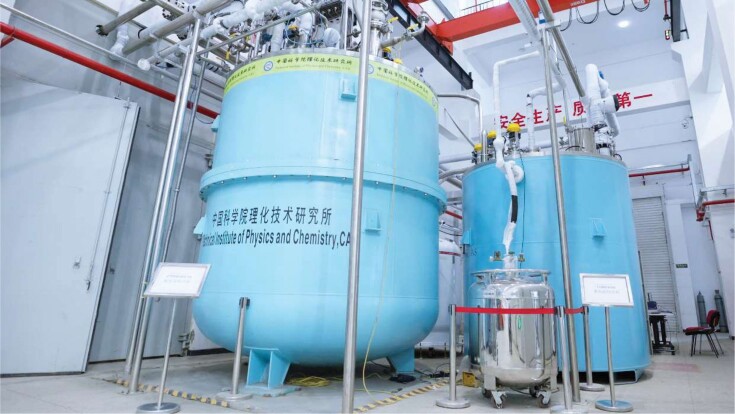
The large-scale cryogenic refrigeration system in the liquid-helium-to-superfluid-helium temperature range. (*Courtesy of the Technical Institute of Physics and Chemistry, Chinese Academy of Sciences*)

## 9 THE MAKING OF A SUPER PEST

A two-decade-long study led by researchers from the Chinese Academy of Agricultural Sciences revealed how whiteflies ‘stole’ a key gene from a plant they were feeding on more than 35 million years ago to become today's super pests. According to a paper published in *Cell*, during a rare event called horizontal gene transfer, those tiny, soft insects acquired a gene that encoded the plant's antidote to toxins it had developed in its own body to poison predatory insects. The research not only provided a crucial piece of evidence for the co-evolution of insects and their host plants, but also indicated how we can use these findings to protect crops more efficiently.

**Figure 9:**
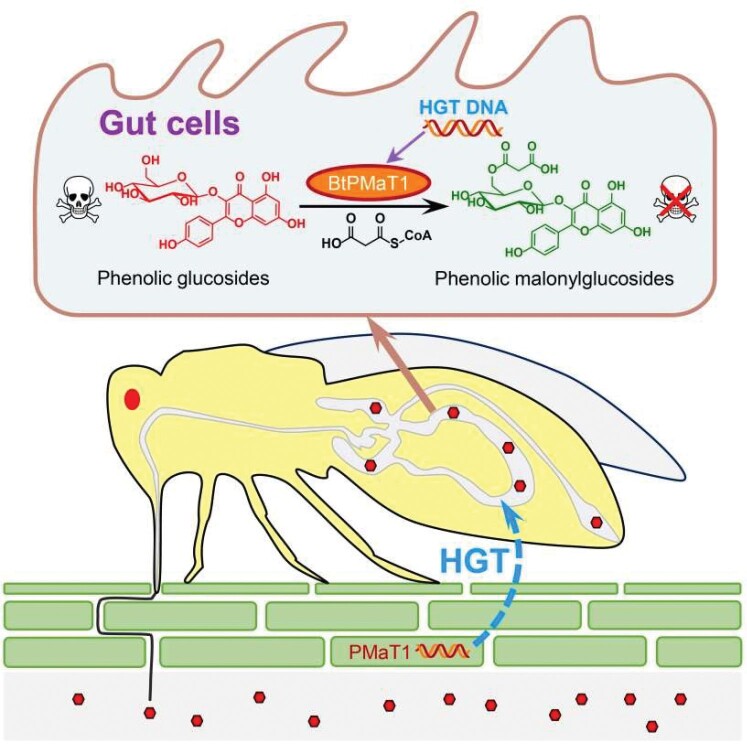
Whiteflies hijack plant gene *BtPMaT1* to neutralize plant toxins. (*Courtesy of the Institute of Vegetables and Flowers, Chinese Academy of Agricultural Sciences*)

## 10 TOWARDS LONG-DISTANCE QUANTUM COMMUNICATION

Quantum relay and transportable quantum memories are two potential solutions for long-distance quantum communication, but both routes rely on the development of high-performance quantum memories. A group from the University of Science and Technology of China in Hefei used rare earth ion-doped crystals to test the world's first quantum repeater segment based on absorptive quantum memories, and achieved the longest, record-breaking optical storage time of 1 hour. These advances helped bring us one step closer to the realization of long-distance quantum communication.

**Figure 10:**
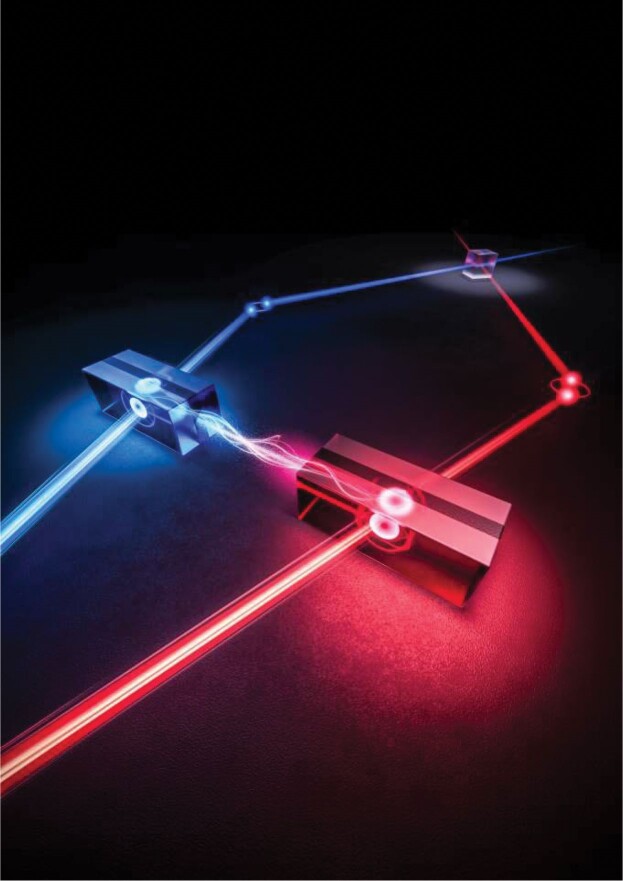
Artistic illustration of the quantum communication. (*Courtesy of the University of Science and Technology of China*)

